# Cerclage is associated with the increased risk of preterm birth in women who had cervical conization

**DOI:** 10.1186/s12884-018-1765-6

**Published:** 2018-07-03

**Authors:** Geum Joon Cho, Yung-Taek Ouh, Log Young Kim, Tae-Seon Lee, Geun U. Park, Ki Hoon Ahn, Soon-Cheol Hong, Min-Jeong Oh, Hai-Joong Kim

**Affiliations:** 1 Department of Obstetrics and Gynecology, Korea University College of Medicine, Seoul, Republic of Korea; 2 The Health Insurance Review and Assessment Service of Korea, Seoul, South Korea; 3 Department of applied statistics, Chung-Ang University, Seoul, South Korea

**Keywords:** Cerclage, Preterm birth, Conization, Preterm premature rupture of membrane

## Abstract

**Background:**

The aim of this study was to determine the effect of cerclage in women who underwent cervical conization.

**Methods:**

Study data were collected from the Korea National Health Insurance Claims Database of the Health Insurance Review and Assessment Service for 2009–2013. Women who had a conization in 2009 and a subsequent first delivery between 2009 and 2013 in Korea were enrolled.

**Results:**

Among the women who had conization in 2009, 1075 women had their first delivery between 2009 and 2013. A cerclage was placed in 161 of the women who were treated by conization. The rate of preterm birth was higher in the women who were treated with cerclage following a conization compared with those without cerclage (10.56 vs 4.27, *p* < 0.01, respectively). The multivariate regression analysis revealed that the women who were treated cerclage following a conization had an increased risk of preterm delivery compared with women without cerclage (odds ratio (OR), 2.6, 95% confidence interval (CI), 1.4–4.9).

**Conclusion:**

Our study showed that cerclage associated with an increased risk of preterm birth and preterm premature rupture of membranes in women who underwent conization. Further studies are required to clarify the mechanism by which cerclage affects the risk of preterm birth.

## Background

Preterm birth is defined as delivery before 37 weeks, and it has been implicated in approximately two thirds of infant deaths [1–3]. Although the infant mortality rate has declined over the past century, it has remained a major health problem. Screening for the risk of preterm labor is not beneficial in the general population. However, a short cervix is one of the poorest predictors of preterm birth [4, 5]. Cervical incompetence is a clinical diagnosis characterized by recurrent, painless cervical dilatation and shortening. Since the 1950s, a cervical cerclage has been a relatively common procedure performed for the treatment of cervical incompetence [6].

A large study from Norway evaluated 15,108 births that occurred in women who had previously undergone cervical conization, and 57,136 who gave birth before conization [7]. The researchers reported the proportion of preterm deliveries in each group, 17.2% versus 6.7%. Even if the cause of cervical incompetence is obscure, previous trauma to the cervix, such as dilatation and curettage or conization, has been implicated. Conization may lead to cervical insufficiency, and cerclage is a treatment option.

The efficacy of prophylactic cerclage for prevention of a preterm birth remains controversial. It has been reported that cervical cerclage cannot only prevent preterm birth but also may be an independent risk factor in women following conization [8]. In a retrospective study of 25 patients with a prior conization who underwent prophylactic cerclage, the treatment did not prevent preterm birth [9].

The aim of this study was to determine the effect of cerclage in women who had cervical conization.

## Methods

We collected the data from the Korea National Health Insurance (KNHI) Claims Database of the Health Insurance Review and Assessment Service (HIRA) for 2009–2013. We have mentioned about KNHI Claims Database [10]. Briefly, 97% of the Korean population is required to enroll in the KNHI program. The remaining 3% of the population is treated under the Medical Aid Program. Thus, this centralized database contained nearly all contents about the occurrence of disease except the disease or treatment that are not covered by insurance. According to the Act on the Protection of Personal Information Maintained by Public Agencies, HIRA possess the claims data by concealing individual identities. The database included an unidentifiable code representing each individual, together with age, diagnosis, and a list of prescribed procedures. Therefore, studies using data from HIRA can be exempt from institutional board reviews. The datasets used and/or analysed during the current study are available from the corresponding author on reasonable request.

All women who underwent conization in 2009 and gave birth during 2009 to 2013 were identified by using the International Classification of Diseases, Tenth Revision (ICD-10) diagnosis and procedure codes. A first pregnancy was linked to conization during the study period. Women who had undergone conization in 2009 and then had their first delivery between 2009 and 2013 were only included in our study. Using procedure code for cerlcage, it was confirmed whether or not cerclage was performed during pregnancy.

To identify women with preterm delivery and preterm premature rupture of membrane (pPROM) from the HIRA database, ICD-10 codes O60.1 for preterm delivery and ICD-10 code O42.x with code for preterm delivery for pPROM were used.

Data about the women’s characteristics, such as age, delivery mode (vaginal delivery or cesarean section), multiple pregnancies (defined as twin or higher-order gestation), and the number of years between conization and delivery, were obtained.

We used the Student’s t-test to compare continuous variables between groups and chi-square test to compare categorical variables. To evaluate risk the risk of preterm delivery, a model of multivariate logistic regression analysis was performed with preterm delivery or pPROM in the second pregnancy.

A *P* value < 0.05 was considered statistically significant. Statistical analyses were performed using SPSS software, version 12.0 (SPSS Inc., Chicago, IL, USA).

## Results

Among the 23,553 women who had conization in 2009, 1075 had their first delivery between 2009 and 2013. Cerclage was placed in 161 of the women who had been treated by conization.

Table 1 shows the basic characteristic of the study population treated by cerclage following conization. Compared to women without cerclage, women with cerclage following conization had higher rates of preeclampsia in the first pregnancy. However, there were no differences in age, rates of multiple pregnancy, or years from conization to delivery between the two groups. The rate of preterm birth was higher in women who underwent cerclage following a conization compared with women without cerclage (10.56 vs 4.27, *p* < 0.01, respectively). The rate of pPROM was also higher in women with cerclage following a conization than in women without cerclage (6.21 vs 2.41, *p* < 0.01, respectively).

**Table 1:** Basic characteristics of the study participants

	Pregnant women		*P*-value
	Without cerclage (*n* = 914)	With cerclage (*n* = 161)	
Age (years)	30.69 ± 2.32	30.79 ± 2.41	0.481
Cesarean section (%)	185 (20.24)	63 (39.13)	< 0.001
Multiple pregnancy (%)	22 (2.41)	6 (3.73)	0.320
Years since delivery from conization (years)	2.14 ± 1.05	2.07 ± 1.03	0.733
Preterm delivery (weeks)	39 (4.27)	17 (10.56)	< 0.001
pPROM (%)	22 (2.41)	10 (6.21)	0.867

pPROM, preterm premature rupture of membranes

The multivariate regression analysis (Table 2) revealed that women with cerclage following a conization had an increased risk of preterm delivery compared with women without cerclage (OR 2.64, 95% CI 1.43–4.87). Women with cerclage following a conization also had an increased risk of pPROM compared with women without cerclage (Table 3) (OR 2.60, 95% CI 1.19–5.64).

**Table 2:** Adjusted odds ratios (OR) and 95% confidence interval (CI) for the risk of preterm birth

	OR	95% CI
Age (years)	1.0	0.9, 1.0
Cerclage (yes)	2.6	1.4, 4.9
Multiple pregnancy (yes)	10.5	4.4, 25.2
Years since delivery from conization (years)	0.9	0.7, 1.2

The model is adjusted for variables in the Table; 95% CI, 95% confidence interval

**Table 3:** Adjusted odds ratios (OR) and 95% confidence interval (CI) for the risk of pPROM

	OR	95% CI
Age (years)	1.0	0.9, 1.1
Cerclage (yes)	2.6	1.2, 5.64
Multiple pregnancy (yes)	5.8	1.9, 18.3
Years since delivery from conization (years)	0.8	0.6, 1.2

The model is adjusted for variables in the Table, 95% CI, 95% confidence interval

## Discussion

In our study, cervical cerclage could not prevent preterm birth among women who had been treated by conization; cerclage was associated with an increased risk of preterm birth compared with that among patients who did not undergo a cerclage. On the basis of previous studies that reported cerclage significantly prevents preterm birth and perinatal mortality and morbidity in women with previous spontaneous preterm birth [11], we hypothesized that cervical cerclage would also prevent preterm birth among women who had been treated by conization. Thus, it is interesting to note that cervical cerclage was actually associated with an increased risk of preterm birth in this study. The reason for this unexpected association is unclear, but there are some plausible explanations. First, suture materials placed in the uterine cervix are a foreign body and can cause inflammation after cervical cerclage. In this study, the rate of pPROM in the preventive cervical cerclage group was higher compared with control group. pPROM is a complex autotoxic disease that involves activation and interaction of the cytokine, MMP, and apoptosis pathways, although it was originally thought to be due to a direct action of bacteria [12]. It has been reported that the 42% of patients with total intra-amniotic inflammation were associated with pPROM even in the absence of intra-amniotic infection, and it was applied to manage pPROM [13]. It can be assumed that cerclage induced cervical inflammation and consequent intraamniotic inflammation, and elevated the preterm birth rate even in the absence of infection. Sakai [13] compared the risk of preterm birth according to cervical mucus interleukin-8 (IL-8) among patients who underwent cervical cerclage for shortening. Their study showed that cerclage may be harmful to patients with elevated cervical mucus IL-8, but that cerclage reduced the risk of preterm delivery in patients with normal cervical mucus IL-8 [13]. Whereas cerclage was effective in patients with a short cervix without cervical inflammation, it was deleterious in patients with inflammation; this finding suggested that cerclage causes preterm delivery by inducing inflammation and chorioamnionitis. Moreover, as pregnant women who had undergone conization were enrolled in this study, cerlcage, repeated trauma may attribute to reduction of tension threshold of cervical tissue, which causes preterm birth. Thus, cervical cerclage itself may influence the development of preterm birth. Another reason for this effect may be that treatment with cerclage was targeted at patients who already had other risk factors of preterm birth, since cervical incompetence can be caused by anatomical defect or uterine abnormities, as well as a short cervix. Moreover, pregnancy outcomes including preterm delivery and pPROM may be different in women who underwent between prophylactic and emergent cerclage. However, as the indication of cerclage and gestational age at cerclage were not available in this study, further studies are needed to evaluate the exact mechanisms by which cerclage may affect development of preterm birth in women who underwent cervical conization.

Our results have implications that might be clinically relevant. In our study, cerclage did not prevent preterm birth; moreover, cervical cerclage was associated with a higher rate of preterm birth. In addition, when compared with the incidence of preterm birth in South Korea, the risk of preterm birth is not higher among women with conization. This finding suggests that in pregnant women who have undergone conization, prophylactic cerclage is not essential for preventing preterm birth; this conclusion is consistent with the results of other recent studies [9, 14]. Rather, our results show that pregnancy after conization is not an absolute indication for prophylactic cerclage, and other risk factors should be considered confounding factors. Several studies have examined the association of cone depth with the risk of preterm birth. The risk of preterm birth increased 6–20% per millimeter of cervical excision [15, 16]. It has been also reported that a depth thicker than 12 mm and larger than 6 cm^3^ carried a 3-fold risk for preterm birth [17]. Because gynecologists who perform conizations and obstetricians providing prenatal care may be different providers in most cases in Korea, clinicians should share the clinical data about conization to avoid unnecessary prophylactic cerclage that could be harmful.

Several limitations should be considered when interpreting the present findings. First, this study was based on insurance claim data in the KNHI Claims Database, which was designed for cost claim issues, not research. Thus, the cause of the preterm births was not available. It has been reported that preeclampsia, fetal distress, small-for-gestational age, and placental abruption were the most common indications for a medical intervention resulting in preterm birth [18]. Therefore, in our study, other potential causes of preterm birth cannot be excluded. However, in this study, the definition of preterm birth was limited to ICD-10 code, O60.1 (preterm spontaneous labor with preterm delivery) to exclude preterm birth by other causes.

Another limitation of our study is that we were not able to access information such as cervical status before conization, and depth of conization, all of which are known factors of preterm delivery [19–21], because these data were not available in the database.

Nevertheless, the strength of the present study lies in the large population-based cohort, with very few patients lost to follow-up. Although there have been several studies about the effectiveness of cerclage on preterm birth, they recruited fewer than 100 patients because of the infrequency of cervical cerclage following conization [9, 14]. By contrast, we evaluated 161 patients who had undergone a cerclage with previous conization and 914 without cerclage. This large study group strengthens the result of our study. Moreover, biochemical characteristics of the cervix have reported to be different according to patient’s parity [22, 23]. Thus, another strength of our study is that we enrolled only women who delivered their first child during the same period to minimize the effect of parity.

## Conclusions

In conclusion, our study revealed that the incidence of preterm birth was not significantly different between the group that underwent conization and the group that did not. Prophylactic cerclage during pregnancy is not necessary for women who have undergone conization. Further studies are needed to add information about the effect of the sonographic cervical length, the time interval between conization and delivery, gestational weeks at cerclage, and cone size, to inform the current guidelines for prophylactic cervical cerclage after conization.

## Abbreviations

HIRA: Health insurance review and assessment service
IL-8: Interleukin-8
KNHI: Korea National Health Insurance
pPROM: Preterm premature rupture of membrane
